# Dengue: A Growing Menace -- A Snapshot of Recent Facts, Figures & Remedies

**Published:** 2013-06

**Authors:** M. K. Bhattacharya, S. Maitra, A. Ganguly, A. Bhattacharya, A. Sinha

**Affiliations:** 1 National Institute of Cholera & Enteric Disease, Kolkata;; 2 ID & BG Hospital, Kolkata

**Keywords:** Dengue Virus, management, vaccine

## Abstract

Dengue is specially owing to inadequate water supply and poor solid waste management , which are favorable for multiplication of the main vectors including the Aedes ageypti coupled with lack of proven anti viral therapy and no proven efficient vaccine .there are many cases of both dengue shock syndrome and dengue haemmorhagic fever making it a major public health burden sending ominous signal resulting both rising morbidity & mortality, deleterious effect on DALY [disability adjusted life year] & QALY [quality adjusted life year] & though it affect all section of society ,still it affect the poor & underprivileged section more, thereby growing menace in public health in general & in developing countries in particular.

## INTRODUCTION

Dengue is one of the most rapidly spreading mosquito-borne viral diseases in the world. The last 50 years has witnessed a 30-fold increase with rapid expansion to new countries and, in the present decade, from urban to rural settings. An estimated 50 million dengue infections occur annually and about 2.5 billion people live in dengue endemic areas (1, 2).

The 2005 World Health Assembly resolution (WHA58.3) on the revision of the International Health Regulations (IHR) has includes dengue a disease that may emerge as a public health emergency of international concern having implications for health security due to disruption and rapid epidemic spread beyond national and international borders (1).

Dengue prevention and control has been implemented through the Bi-regional Dengue Strategy (2008-2015) of the WHO South-East Asia and Western Pacific regions. This consists of following six elements:

- Dengue surveillance;
- Case management;
- Outbreak response;
- Integrated vector management;
- Social mobilization and communication for dengue, and
- Dengue research (a combination of both formative and operational research) (1).

### History

The first case of probable dengue fever (DF) was recorded in a Chinese medical encyclopedia from the Jin Dynasty (265–420 AD) which referred to as “water poison” associated with flying insects. There have been descriptions of epidemics in the 17th century, but the most plausible early reports of dengue epidemics occurred between 1779 and 1780, when an epidemic swept through Asia, Africa and North America. From that time until 1940, epidemics were infrequent (2, 3).

In 1906, transmission by the *Aedes* mosquitoes was confirmed, and in 1907 dengue was the second disease (after yellow fever) that was shown to be caused by a virus. Further investigations by John Burton Cleland and Joseph Franklin Siler completed the basic understanding of dengue transmission (2, 3).

Dengue fever in India was first reported in 1946 and many small outbreaks occurred after that in different parts of country, like in Vishakhapatnam in 1969 and Jalore, Rajasthan in 1985, but first dengue hemorrhagic fever (DHF) case was reported in Kolkata back in 1964, and Delhi experienced major outbreak in 1996 (22, 23). During an investigation of an outbreak of unknown fever in siliguri, district Darjeeling, in oct-nov 2005 dengue fever cases were confirmed (24).

## EPIDEMIOLOGY

There are probably tens of millions of cases of dengue each year, and at least five hundred thousand cases of DHF with a mortality of about five per cent in most countries. The vast majority of cases, nearly 95%, are children less than 15 years of age. Clearly, most widespread mosquito-borne disease is a major public health problem (2).

## BURDEN OF DISEASE – A PUBLIC HEALTH CONCERN

Dengue inflicts a significant health, economic and social burden on the populations of endemic areas. Globally the estimated number of disability-adjusted life years (DALYs) lost to dengue in 2001 was 528 days (8).

The number of case annually reported to WHO ranged from 0.4 to 1.3 million in the decade of 1996–2005 with an average episode represented 14.8 lost days for ambulatory patients and 18.9 days for hospitalized patients. The overall cost of a non-fatal ambulatory case averaged US$ 514, while the cost of a non-fatal hospitalized case averaged US$ 1491. Children are at a higher risk of severe dengue (17).

Dengue afflicts all levels of society but the burden may be higher among the poorest who lives in communities with (18).

### Dengue in WHO South-East Asia Region

Since 2000, dengue has spread to newer areas and also has increased in the previously affected areas of the region. In 2003, eight countries -- Bangladesh, India, Indonesia, Maldives, Myanmar, Sri Lanka, Thailand and Timor-Leste -- reported dengue cases. In 2004, Bhutan reported the country’s first dengue outbreak. In 2005, WHO’s Global Outbreak Alert and Response Network (GOARN) responded to an outbreak with a high case-fatality rate (3.5%) in Timor-Leste. In November 2006, Nepal reported indigenous dengue cases for the first time. The Democratic Peoples’ Republic of Korea is the only country of the South-East Region that has no reports of indigenous dengue.

Recurrent DF epidemics are increasing in its frequency and in-country geographic expansion is occurring in Bangladesh, India and Maldives in the deciduous dry and wet climatic zone with multiple virus serotypes. Sri Lanka experienced the largest epidemic of DF/DHF with as many as 70,000 cases and almost 600 deaths during 2009-10 and 25,303 cases and 169 deaths, respectively in 2011 (21).

First dengue fever case in West Bengal (WB) was reported during 1963 and also the first Dengue haemorrhagic fever was reported in 1963-64 (22).

There is a clear seasonal trend in Kolkata with majority of the cases during post-monsoon season, especially from September onwards with exceptional cases all over the year. Due to the heavy burden of this disease, it is important to initiate feasible strategies to control and eradicate the DF (25).

### District wise distribution of DENGUE cases in 2010 in west Bengal (Figure 1)

Both dengue virus development and female Aedesaegypti mosquito biting rates are sensitive to temperature and the potential risk increases to approximate 31-47% due to an approximate 10°C rise in temperature. Data noted that the case number over the last three years has decreased and the mortality rate is very low. And that can be attributed to both global warming and inadequate surveillance system and health informatics at many part. However, the detailed district reports suggests that Kolkata, North 24 Parganas and South 24 Parganas still remains endemic with the number of case being more than 50 indicating urbanization may be an important parameter for the transmission as well as occurrence of the disease. Apart from these endemic zones, a close monitoring of the district report over the last three years reveals that there is a sharp rise in the case numbers especially in the district of Bankura and Hooghly in 2010. This may be a result of global warming available in health statistics of west bengal government health department website.

**Figure 1:**
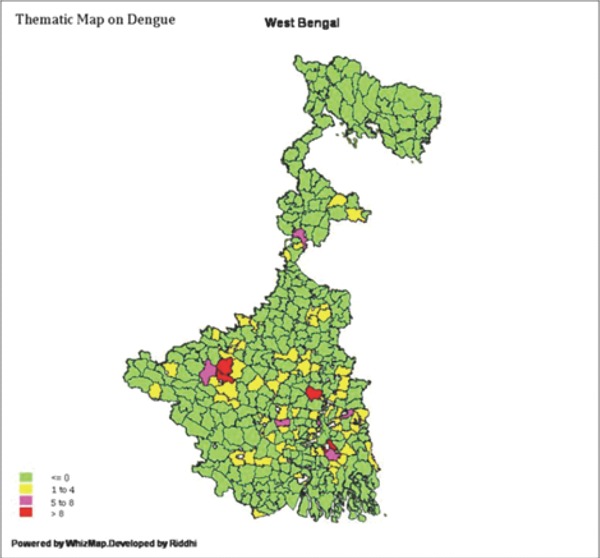
District wise case and deathdistribution available in HEALTH STATISTICS section of west Bengal government Health department website.

## DENGUE VIRUS

Dengue virus is a mosquito-borne single positive-stranded RNA virus of the family *Flaviviridae*; of the genus *Flavivirus* (this sentence already presented in the first paragraph). Its genome is about 11000 bases that codes for three structural proteins, capsid protein C, membrane protein M, envelope protein E; seven nonstructural proteins, NS1, NS2a, NS2b, NS3, NS4a, NS4b, NS5; and short non-coding regions on both the 5’ and 3’ ends (27).

All the four serotypes, referred to as DENV-1, DENV-2, DENV-3 and DENV-4.can cause the full spectrum of disease. Infection with one serotype is believed to produce lifelong immunity to that serotype but only short term protection against the others. The severe complications on secondary infection occurs particularly if someone previously exposed to serotype DENV-1 then contracts serotype DENV-2 or serotype DENV-3, or if someone previously exposed to type DENV-3 then acquires DENV-2 (1, 10, 29-31).

Suggest to describe further on the antibody-dependent mechanism of dengue infection.

### Entomology

Dengue virus is transmitted to humans through the bites of infected *Aedes* mosquitoes, principally *Aedes aegypti*. This mosquito is a tropical and subtropical species widely distributed around the world than the other species such as *Aedes albopictus, Aedes polynesiensis* and several species of the *Aedes scutellaris* complex (1, 11, 28).

### Transmission of virus

Humans are the main amplifying host of the virus. Dengue virus circulating in the blood of viraemic humans is ingested by female mosquitoes during feeding. The virus then infects the mosquito mid-gut and subsequently spreads systemically over a period of 8 to 12 days. After this extrinsic incubation period, the virus can be transmitted to other humans during subsequent probing or feeding. The extrinsic incubation period is influenced in part by environmental conditions, especially the ambient temperature. Thereafter, the mosquito remains infective for the rest of its life. *Aedes aegypti* is one of the most efficient vectors for arboviruses because it is highly anthropophilic, frequently bites several times before completing oogenesis, and thrives in close proximity to humans. Vertical transmission (transovarial transmission) of dengue virus has been demonstrated in the laboratory but rarely in the field (4, 6, 11).

### Dengue Case Definitions

**According to WHO:**

1. **Definition of probable case of dengue fever**
  Person who live or travel t dengue endemic area and have two or more symptom along with fever:
    - Myalgia
    - Arthralgia
    - Rash
    - Hemorrhagic Manifestations
    - Retro-orbital pain
    - Headache
    - Leucopenia
2. **Definition for CONFIRMED cases of Dengue Fever:**
  A suspected case of dengue fever having either:
    - NS1 Antigen ELISA Test reactive (in case of 2-4 days duration of fever), OR
    - MAC ELISA IgM Test reactive (in case of fever 5 days or more), OR
    - RT-PCR positive.
3. **Case Definition for Dengue Hemorrhagic Fever:**
  All must be present with:
    - Fever, or recent history of acute fever;
    - Thrombocytopenia (100,000 mm/cu.mm or less);
    - Haemorrhagic tendencies, as evidenced by at least one of the Following:
      - Positive tourniquet test;
      - Petechiae, ecchymoses or purpura;
      - Bleeding from mucosa, gastrointestinal tract, injection sites, or others.
    - Plasma leakage due to increased capillary permeability as manifested by at least one of the following:
      - Hematocrit on presentation that is >20% above average for that age and population.
      - >20% drop in haematocrit following treatment.
      - Commonly associated signs of plasma leakage: Ascites Hypoproteinemia, Pleural effusion.
    - Case Definition for Dengue Shock Syndrome:
  All four criteria above plus evidence of circulatory failure manifested by all of the following:
    - Rapid and weak pulse,
    - Narrow pulse pressure (20 mmHg or less) or hypotension for age, and
    - Cold clammy skin and altered mental status.

### Disease Course

Dengue fever has an unpredictable course. Most patients have a febrile phaselasting 2-7 days. This is followed by a critical phase which is ofabout 2-3 days duration. During this phase, the patient is afebrile, and is at risk of developing DHF/DSS which may prove fatal if prompt and appropriate treatment is not provided. Since haemorrhage and or shock can occur rapidly, arrangements for rapid and appropriate treatment should be always available. By doing this, the case fatality rate can be substantially reduced.

The fever typically follows the following stages in progression:

**Table:** 

**Febrile Phase** Dehydration; high fever may cause neurological disturbances and febrile seizures in children.
**Critical Phase** Shock from plasma leakage; severe hemorrhage; organ impairment.
**Recovery Phase** Hypervolemia (only if intravenous fluid therapy has been excessive &/or has extended into this period).

Courtesy: WHO 2009 guideline (1, 12-14).

## MANAGEMENT

### Treatment of DF and DHF

#### Febrile Phase

In the early febrile phase, it is not possible to distinguish DF from DHF.

The treatments during the febrile phase are the same, i.e. symptomatic and supportive:

- Rest.
- Paracetamol (not more than 4 times in 24 hours) according to age for fever above 39degC.
- Not to give Aspirin or Brufen. Aspirin can cause gastritis and/or bleeding. In children, Reye’s syndrome (encephalopathy) may be a serious complication.
- Not to give antibiotics as these do not help.
- Oral rehydration therapy is recommended for patients with moderate dehydration caused by vomiting and high temperature.
- Food should be given according to appetite.
- All dengue patients has to be observed for complications for at least two days after recovery from fever. Life threatening complications tend to occur during this phase. Patients and households should be informed that bleeding into the skin or from the nose or gums, passage of black stools, severe abdominal pain, sweating, and cold skin are danger signs. If any of these signs is noticed, the patient should be taken to the hospital.

Depending on the clinical manifestations and other circumstances, patients may be sent home (Group A), be referred for in-hospital management (Group B), or require emergency treatment and urgent referral (Group C).

### Group A – patients who may be sent home

These are patients who are able to tolerate adequate volumes of oral fluids and pass urine at least once every six hours, and do not have any of the warning signs, particularly when fever subsides.

Indications for domiciliary treatment:

- No Hypotension;
- No Tachycardia;
- No Narrowing of Pulse Pressure;
- No Bleeding;
- Thrombocytes > 100,000 cells/cu.mm.

Treatment:

1. ORS;
2. Paracetamol;
3. To instruct the care-givers that the patient should be brought to hospital immediately in case of any warning signs. Patient should come for follow-up after 24 hours for evaluation.

### Group B – patients who should be referred for in-hospital management

Patient to be referred or to attend health care centre for close observation, particularly as they approach the critical phase, particularly for the following signs and symptoms:

- No clinical improvement;
- Deterioration around the time of defervescence;
- Severe abdominal pain;
- Persistent vomiting;
- Cold and clammy extremities;
- Lethargy or irritability/restlessness,
- Bleeding (e.g. black stools or coffee-ground vomiting);
- Not passing urine for more than 4–6 hours;
- Restlessness, Seizures, Excessive crying (young infant), altered sensorium.

Treatment:

1. Obtain a reference haematocrit before fluid therapy. Give only isotonic solutions such as 0.9% saline/Ringer’s lactate/Hartmann’s solution;
2. Reassess the clinical status and repeat the Haematocrit;
3. Give the minimum intravenous fluid volume required to maintain good perfusionand urine output of about 0.5 ml/kg/hr. Intravenous fluids are usually needed for only 24–48 hours.

### Group C – patients who require emergency treatment and urgent referral

When they have:

- Severe plasma leakage leading to dengue shock and/or fluid accumulation with respiratory distress;
- Severe haemorrhages;
- Severe organ impairment (hepatic damage, renal impairment, cardiomyopathy, encephalopathy or encephalitis).

### Methods of vector control

#### Environmental management

##### Environmental modification

*Environmental manipulation*

*Changes to human habitation or behavior*: Installing mosquito screening on windows, doors and otherentry points, and using mosquito nets while sleeping during daytime:

- Improvement of water supply and water-storage systems;
- Mosquito-proofing of water-storage containers;
- Solid waste management;
- Street cleansing;
- Building structures.

### Chemical control: larvicides

Aedes aegypti breeds in clean water used for household purposes. Hence, larvicides, which are safe, without any odour or colour, have residual effect with low toxicity and do not pose any public health hazard should be used.

Temephos, an organophosphate compound meets all the above mentioned requirements and this insecticide is being used under the public health programme. The recommended dose for application of Temephos (50 EC) is 1 ppm (1 mg per liter of water) (15).

### Chemical control: adulticides

The following methods are recommended for the control of adult Ae aegypti mosquitoes:

- Pyrethrum spray: It may be used in indoor situations as space spray at a concentration of 0.1%-0.2% @ 30-60 ml/1000 cu. ft. Commercial formulation of 2% pyrethrum extract is diluted with kerosene in the ratio of one part of 2% pyrethrum extract with 19 parts of kerosene (volume/volume). Thus, one litre of 2% pyrethrum extract is diluted by kerosene into 20 litres to make 0.1% pyrethrum formulation (ready-to-spray formulation). After dilution, pyrethrum extract is sprayed with Flit pump or hand operated fogging machine fitted with microdischarge nozzle.
- Malathion fogging or Ultra Low Volume (ULV) spray: For fogging Commercial formulation of Malathion is diluted with diesel in the ratio of one part of Technical Malathion with 19 parts of diesel (volume/volume). In application of ULV, minimum volume of liquid insecticide formulation is applied per unit area. That is, the insecticide is broken down into small droplets of a volume median diameter (VMD) of 40-80 microns with an objective of producing a cloud of insecticide droplets that remain suspended in air for an appreciable time and driven under the influence of wind. This provides maximum effectiveness against target vectors. Since no diluents is used, the technique is more cost-effective than thermal fogging but it does not generate a visible fog. Most organo-phosphorus insecticides in their technical form can be applied as ULV spray. Under the public health programme.

### ULV spray

(Fogging) is undertaken by using 95% or pure technical malathion. The ground equipment mostly used for ULV spray includes portable motorized knapsack blowers and cold aerosol generators (15).

### Biological control

Only certain species of larvivorous fish and predatory copepods (Copepoda: Cyclopoidea) – small freshwater crustaceans – have proved effective in operational contexts.

*Insecticide-treated materials (ITMs), typically deployed as insecticide-treated bednets & insecticide-treated window curtains

### *Lethal Ovitraps

Scope for further research

Further research efforts is required to prevent and treat dengue include various means of:

1. Vector control;
2. Vaccine development, and
3. Antiviral drugs

### Vector control

Guppy (*Poecilia reticulata*) or copepods in standing water to eat the mosquito larvae. Attempts are ongoing to infect the mosquito population with bacteria of the *Wolbachia* genus, which makes the mosquitoes partially resistant to dengue virus

### Vaccine development

There are ongoing programs working on a dengue vaccine to cover all four serotypes. One of the concerns is that a vaccine could increase the risk of severe disease through antibody-dependent enhancement. The ideal vaccine is safe, effective after one or two injections, covers all serotypes, does not contribute to ADE, is easily transported and stored, and is both affordable and cost-effective. As of 2009, a number of vaccines were undergoing testing. It is hoped that the first products will be commercially available by 2015.

### Antiviral drug

Discovery of the structure of the viral proteins may aid the development of effective drugs. There are several plausible targets. The first approach is inhibition of the viral RNA-dependent RNA polymerase (coded by NS5), which copies the viral genetic material, with nucleoside analogs. Secondly, it may be possible to develop specific inhibitors of the viral protease (coded by NS3), which splices viral proteins. Finally, it may be possible to develop entry inhibitors , which stop the virus entering cells, or inhibitors of the 5′ capping process, which is required for viral replication (29-31).

### WHO response

WHO responds to dengue in the following ways:

- Supports countries in the confirmation of outbreaks through its collaborating network of laboratories;
- Provides technical support and guidance to countries for the effective management of dengue outbreaks;
- Provides training on clinical management, diagnosis and vector control at the regional level with some of its collaborating centres;
- Formulates evidence-based strategies and policies;
- Develops new tools, including insecticide products and application technologies; gathers official records of dengue and severe dengue from over 100 Member States; publishes guidelines and handbooks for dengue prevention and control for Member States.

## CONCLUSIONS

Dengue fever continues to be a public health menace and a global threat. As of now, it is endemic and appears in opportune time and places as an epidemic in almost the entire tropics. Judicious and appropriate use of available interventions should be commenced as we wait for newer vaccines, antiviral drugs and improved diagnostics. The future challenge would be as to how we deploy these newer tools. A global strategy aimed at increasing the capacity for surveillance and outbreak response, changing behaviours and reducing the disease burden using integrated vector management in conjunction with early and accurate diagnosis has been advocated. Antiviral drugs and vaccines that are currently under development could also make an important contribution to dengue control in the future.
